# Pathophysiology of cutaneous lupus erythematosus

**DOI:** 10.1186/s13075-015-0706-2

**Published:** 2015-08-10

**Authors:** Jordan C Achtman, Victoria P Werth

**Affiliations:** Philadelphia VA Medical Center, 3900 Woodland Avenue, Philadelphia, PA 19104 USA; Department of Dermatology, Perelman Center for Advanced Medicine, 3400 Civic Center Boulevard, Philadelphia, PA 19104 USA

## Abstract

The pathophysiology of cutaneous lupus erythematosus (CLE) encompasses the complex interactions between genetics, the environment, and cells and their products. Recent data have provided enhanced understanding of these interactions and the mechanism by which they cause disease. A number of candidate genes have been identified which increase the risk of developing CLE. Ultraviolet radiation, the predominant environmental exposure associated with CLE, appears to initiate CLE lesion formation by inducing apoptosis, precipitating autoantigen presentation, and promoting cellular production of specific cytokines. Autoantibodies are a well-known entity in CLE, but their exact role remains unclear. Finally, cells ranging from native skin cells to innate and adaptive immune cells produce cytokines and other molecules and play specific roles in lesion formation and perpetuation. Native skin cells implicated in CLE include keratinocytes and endothelial cells. Innate immune cells crucial to CLE pathophysiology include dendritic cells and neutrophils. The primary adaptive immune cells thought to be involved include Th1 cells, Th17 cells, cytotoxic T cells, and invariant natural killer T cells. Though the pathophysiology of CLE has yet to be fully characterized, current research provides direction for future research and therapies.

## Introduction

Cutaneous lupus erythematosus (CLE) is an autoimmune disease with various subsets and wide-ranging clinical manifestations. The primary CLE subsets are discoid lupus erythematosus (DLE), subacute cutaneous lupus erythematosus (SCLE), and acute cutaneous lupus erythematosus (ACLE). While the skin manifestations of lupus erythematosus (LE) have been described for many years, the pathophysiology of CLE remains to be fully characterized. Recent increased recognition of, and interest in, this disease has resulted in enhanced understanding of the etiology of CLE. The initiation and perpetuation of CLE involves genetic risk factors, environmental exposures, and cellular components of the skin and the innate and adaptive immune systems [1].

## Genetics

### Major histocompatibility complex

Certain major histocompatibility complex (MHC) class I and II alleles that may confer susceptibility to CLE include HLA B8, DR3, DQA1, and DRB1. HLA DR3 and DR2 are associated with positivity for Ro-SSA autoantibodies and SCLE. Specific alleles of HLA DQA1 and DRB1 appear to be associated with DLE [2, 3]. MHC polymorphisms that increase susceptibility to disease may do so by allowing escape of autoreactive T lymphocytes from negative selection in the thymus. This failed purging of autoreactive cells may be mediated by decreased affinity of specific MHCs for autoreactive T-cell receptors, the interaction of which is vital to this selection process. Furthermore, these MHC polymorphisms may have decreased ability to select for regulatory T cells (Tregs) that can increase self-tolerance [4].

### Complement

A *C1QA* single-nucleotide polymorphism (SNP) has been found to be highly associated with SCLE and lower C1 serum protein levels. Congenital C1q deficiency is highly associated with photosensitive systemic lupus erythematosus (SLE). While the mechanism is unknown, C1q may be involved in clearance of post-apoptotic immunogenic material. However, studies in C1q-deficient mice did not show a difference in clearance of apoptotic keratinocytes (KCs) after ultraviolet (UV) radiation compared with wild type. Chronic UV exposure did not result in production of autoantibodies either in C1q-deficient mice [5]. Other complement components may be involved in CLE pathogenesis. Hereditary deficiencies in C2 and C4 have been found to be associated with CLE-like skin lesions. This may be related to failure of fixation of immune complexes. There is, however, little evidence for either of these proposed mechanisms [6, 7].

### Tumor necrosis factor-α

Tumor necrosis factor (TNF)-α, a primary cytokine in inflammatory cascades, promotes release of secondary cytokines and recruitment of immune cells, ultimately leading to tissue destruction. It may also promote presentation of autoantigens at the cell surface and subsequent autoreactivity [8, 9]. The TNF-α promoter polymorphism -308A is associated with SCLE but not DLE. In addition, −308A polymorphism is associated with HLA-DR3. The promoter polymorphism appears to increase transcription *in vitro* when transfected cells are exposed to UVB radiation in the presence of interleukin (IL)-1α, a photoinduced cytokine. The difference may be due to differential binding of transcription factors at promoter variants [8].

### *TYK2*, *IRF5*, and *CTLA4*

A panel of SNPs previously implicated in SLE risk were analyzed for CLE risk. *TYK2*, *IRF5*, and *CTLA4* are associated with CLE while *GIMAP5*, *FCGR2A*, and *PDCD1* are not. However, clinical characteristics were not associated with specific genotypes of *TYK2* or *IRF5. TYK2* is associated with DLE. TYK2, a Janus kinase, binds to the interferon (IFN)-α receptor 1 and is involved in cytokine signaling. Activation of TYK2 leads to expression of IFN-regulated genes [10].

*IRF5* is associated with DLE and SCLE. IRF5 is a transcription factor which regulates type I IFNs and has broad effects on the immune system. Certain IRF5 variants may cause prolonged inflammatory response and disrupt immune tolerance. Variants causing increased expression of IRF5 likely lead to increased production of type I IFNs and excessive pro-inflammatory response. Type I IFNs recruit T cells into skin lesions of patients with CLE. Increased expression of IRF5 is also seen in UV-irradiated skin, which supports the role of this transcription factor in the pathophysiology of CLE [10].

*CTLA4* is also associated with DLE. CTLA4 regulates T-cell activation and survival. Variants in *CTLA4* may prevent appropriate limitation of T-cell response in inflammation [10].

### *ITGAM*

*ITGAM* polymorphisms are associated with DLE and SLE, conferring a greater risk for DLE than for SLE. The risk for DLE is independent of the risk for systemic involvement. *ITGAM* encodes the α-chain of α_M_β_2_-integrin, a cell surface receptor involved in inflammation. α_M_β_2_-integrin is found on the surface of neutrophils, macrophages, and dendritic cells and is involved in leukocyte adhesion, phagocytosis, and apoptosis. Its ligands include complement 3 cleavage fragment and ICAM-1. The mechanism of increased risk for DLE/SLE with certain variants may be due to abnormal removal of apoptotic cells and impaired phagocytosis, leukocyte trafficking, and immune regulation [11].

### *TREX1*

Familial chilblain lupus is a variant of CLE characterized by painful, ulcerating, and inflammatory acral papules and nodules. The lesions are precipitated by cold and wet exposure. The condition is associated with arthralgias. A heterozygous missense mutation in *TREX1* was found to be responsible for familial chilblain lupus. Familial chilblain lupus is thus inherited in an autosomal dominant fashion. *TREX1* is a gene encoding the 3’-5’ repair exonuclease 1, which catalyzes the excision of nucleoside monophosphates from 3’ termini of DNA. TREX1 functions as a homodimer, the enzymatic activity of which is decreased in the heterozygous state. The heterozygous missense mutation does decrease TREX1 activity but does not have a dominant-negative effect. TREX1 appears to be important in caspase-independent apoptosis. Specifically, TREX1 is important for granzyme A-mediated cell death, which is impaired in the heterozygous state. Defective apoptosis may lead to loss of self-tolerance and autoreactive immune cells. Furthermore, given that TREX1 functions to degrade DNA, the missense mutation may be responsible for defective DNA degradation during apoptosis, leading to abnormal clearance of DNA and induction of autoimmunity [12, 13]. However, while in some instances TREX1 may promote apoptosis, TREX1 may actually play a role in preventing apoptosis in cells exposed to genotoxic stress from UV light and chemical genotoxins as demonstrated by Christmann *et al.* [14]. These divergent findings indicate the varied and setting-dependent function of TREX1.

Recent data have shed light on additional aspects of *TREX1* in autoimmune disease. TREX1-deficient mice, known to develop type I IFN-dependent autoimmunity, display upregulation of type I IFN-dependent genes in the skin and cutaneous deposition of immunoglobulins and complement [15]. TREX1 also appears to regulate macrophage response to pro-inflammatory stimuli. In TREX1 deficiency, macrophages increase production of TNF-α and type I IFNs, increase presentation of the costimulatory molecule CD86 and antigens to CD4+ T cells, and show decreased ability to clear apoptotic cells [16]. Taken together, these findings suggest mutations in and dysregulation of *TREX1* have the potential to lead to inappropriate activation of the immune system and development of autoimmunity.

## Ultraviolet radiation

### Photosensitivity

Photosensitivity in CLE varies widely in the literature. Photosensitivity has been found to range from 27 % to 100 % in SCLE and from 25 % to 90 % in DLE. These varied results are accounted for by differences in patient perception of photosensitivity as well as differences in techniques used in photoprovocation studies. Patient-reported history of photosensitivity does not predict a positive reaction to phototesting. Skin lesions cannot always be reproduced during photoprovocation tests, and photosensitivity does not necessarily indicate the formation of photoinduced lesions. Rather, photosensitivity encompasses a wide range of symptoms in response to light [17].

### Apoptosis

Apoptosis is a normal part of KC development. Under normal conditions, apoptosis occurs in the granular cell layer of the epidermis at the interface with the stratum corneum. External factors, however, are able to induce premature apoptosis of KCs. Basilar cells are more resistant to induction of premature apoptosis, while suprabasilar KCs much more readily undergo premature apoptosis under certain conditions. It has been shown that UV light can cause KC apoptosis by multiple mechanisms, including generation of reactive oxygen species, DNA damage, and activation of Fas and FasL, among others [9].

UV radiation has been shown to induce specific patterns of KC apoptosis in various subsets of CLE. Norris *et al*. [18] demonstrated an elevated number of apoptotic cells in the basal zone of the epidermis of DLE lesions. In contrast, SCLE lesions showed increased apoptotic cells in the suprabasilar epidermis. Normal skin showed no apoptosis. Kuhn *et al.* [19] found an abnormal increase in the number of apoptotic cells in UV-induced CLE lesions, which might reflect an accumulation due to impaired clearance. The investigators hypothesized that this may lead to secondary necrosis of the uncleared apoptotic cells and the release of pro-inflammatory molecules leading to tissue inflammation and autoimmunity. The etiology of impaired clearance is unclear. However, decreased phagocytosis of apoptotic material by macrophages derived from SLE patients has been demonstrated, which may be the case in CLE as well [20]. Chen *et al.* [21] demonstrated that autoantibodies to certain macrophage receptors may be responsible for decreased clearance of apoptotic material. In contrast to these findings, however, Reefman *et al.* [22, 23] found no increase in apoptosis induction, clearance of apoptotic cells, or secondary necrosis in UVB-irradiated skin of SLE patients when compared with healthy controls. This disparate finding may be due to differences in the technique used to detect apoptotic cells. In SLE patients, the investigators also found inflammatory lesions in the vicinity of apoptotic cells and increased numbers of macrophages. They hypothesized that macrophage clearance of apoptotic material may represent a pro-inflammatory process accounting for the difference in response to UVB radiation despite no difference in rate of apoptosis or apoptotic cell clearance.

### Autoantigens and autoantibodies

**Fig. 1:**
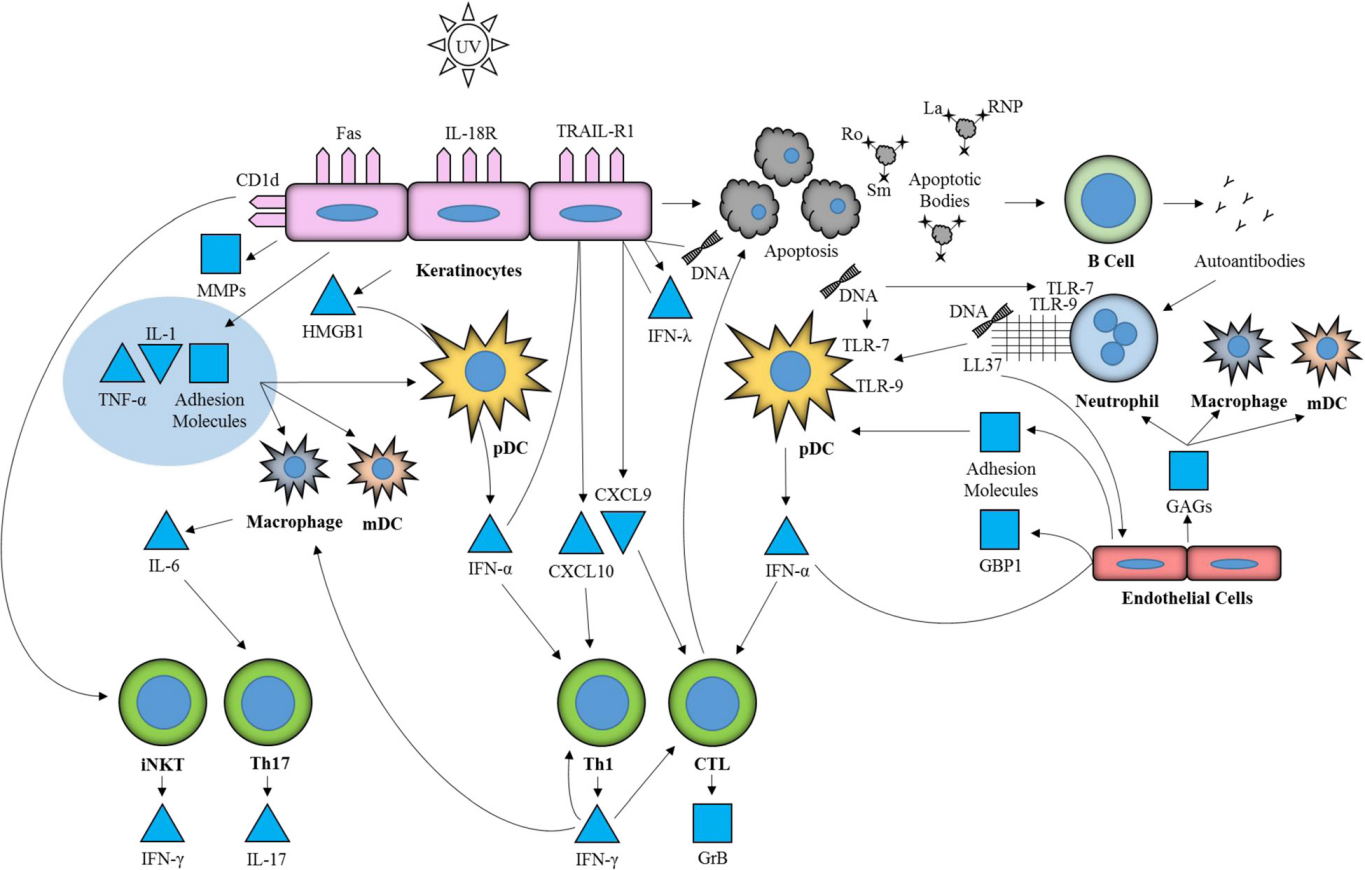
Keratinocytes (KCs) produce cytokines and pro-inflammatory molecules in response to ultraviolet (UV) radiation and other damage. These cytokines recruit and activate members of the innate immune system, including macrophages, myeloid dendritic cells (mDCs), and plasmacytoid dendritic cells (pDCs). Endothelial cells also recruit cells from the innate immune system through adhesion molecules and glycosaminoglycans (GAGs). Endogenous DNA from apoptosis and neutrophil extracellular traps enhance cytokine production by pDCs. pDCs produce interferon (IFN)-α, which recruits members of the adaptive immune system, including Th1 and cytotoxic T cells (CTLs). Th1 cells produce IFN-γ that activates macrophages and CTLs and upregulates its own production. CTLs prime KCs and other cells for apoptosis through granzyme B (GrB), which activates caspases in the target cells. KCs recruit invariant natural killer T cells (iNKTs) through enhanced expression of the antigen-presenting molecule CD1d. iNKTs produce IFN-γ among other cytokines. Various immune cells promote differentiation and activity of Th17 cells through the production of IL-6. Th17 cells produce IL-17. Activity of these cells and cytokines lead to lesion formation and perpetuation characteristic of cutaneous lupus erythematosus. MMP, matrix metalloproteinase; TLR, Toll-like receptor

UV radiation-induced apoptosis causes relocalization of autoantigens [18]. Lefeber *et al*. [24] found that UV radiation induces relocalization to the cell surface of SSA/Ro, SSB/La, RNP, and Sm. Antibodies to these antigens are common in CLE. Subsequent studies demonstrated that Ro and La relocalize to apoptotic bodies of UV-irradiated KCs undergoing programmed cell death (Fig. 1) [25]. Findings by Lawley *et al*. [26] suggest apoptosis, however, may not be necessary for presentation of these autoantigens at the cell surface after exposure to UV light.

### Cells and cytokines

UV radiation causes not only apoptosis and autoantigen presentation but also recruitment of immune cells and production of cytokine cascades. UV light is clearly responsible for the generation of inflammation in both normal skin and skin from patients with CLE. The mechanism of increased susceptibility to inflammation and damage by UV radiation is still far from clear. Skin infiltration by leukocytes and other immune cells in response to UV light are crucial to the development of CLE lesions. T lymphocytes are the predominant cell type found in lesions, though plasmacytoid dendritic cells (pDCs) and myeloid dendritic cells are also increased. UV radiation mediates the production and release of cytokines and chemokines, promoting inflammation and recruitment of immune cells [27, 28].

More specifically, UVB induces KC release of IL-1 and TNF-α, primary cytokines in the inflammatory cascade (Fig. 1). UVB radiation works synergistically with IL-1α to increase production of TNF-α. TNF-α also appears to increase its own production in an autocrine manner. These cytokines subsequently mediate the release of secondary cytokines. This cytokine cascade, recruitment of immune cells, inflammation, and tissue destruction ultimately result in the photoinduced lesions of CLE [9, 29].

## Autoantibodies

The role of autoantibodies in CLE is uncertain. Whether these autoantibodies play a role in the pathogenesis of the disease or are merely non-acting byproducts of immunity gone awry is unclear. These antibodies, however, may prove to be valuable as prognostic indicators.

### Ro, La, Sm, RNP

Various autoantibodies have been found in patients with CLE. The role of these autoantibodies in CLE is unknown. Anti-Ro/SSA and anti-La/SSB are such autoantibodies closely associated with CLE. Biazar *et al*. [30] found that anti-Ro/SSA antibodies were detected in 72.1 % of patients with SCLE, 47.4 % of patients with ACLE, and 22 % of patients with DLE. It was also noted that patients with ACLE and positive for anti-Ro/SSA antibody reported significantly more photosensitivity than patients who were negative for the antibody; however, photoprovocation did not reveal a difference in photosensitivity between these groups. Anti-La/SSB antibodies were detected in 36.2 % of patients with SCLE, 27.5 % of patients with ACLE, and 7.0 % of patients with DLE. No difference was found in reported photosensitivity or photoprovocation for patients positive or negative for anti-La/SSB.

While the role of autoantibodies is unknown, Li *et al*. [31] and Wasicek and Reichlin [32] were able to demonstrate clustering of clinical manifestations with common antibodies in patients with various subsets of LE, including cutaneous and systemic. The patients with both anti-Ro/SSA and anti-La/SSB showed the highest prevalence of discoid rash, photosensitivity, and hematological involvement. Patients with anti-RNP, anti-Sm, and anti-aPL antibodies demonstrated high prevalence of malar rash among other clinical manifestations. Both of these groupings negatively correlated with renal involvement. These studies also confirmed previous reports that anti-Ro/SSA alone is a risk factor for nephritis [31, 32]. While these findings do not define a role for these antibodies in the pathogenesis of cutaneous lesions, they do demonstrate that antibodies can be used as prognostic factors.

### Immunoglobulin isotype

A recent study by Jost *et al.* [33] found that immunoglobulin (Ig)G, IgM, and IgA anti-nuclear antibodies are elevated in SLE compared with DLE. The ratio of IgG to IgM is also increased in SLE compared with DLE. The authors suggest that the higher ratio represents increased class-switching and enhanced humoral response in SLE compared with DLE. Additionally, IgM anti-nuclear antibodies may help to counteract the production of IgG anti-nuclear antibodies and their deleterious effects by decreasing dendritic cell activation and acting as competitive inhibitors by targeting the same nuclear antigens. This may ultimately prevent systemic spread of disease in DLE.

## Cells and their products

### Keratinocytes

KCs are the primary apoptotic cells in CLE. As stated earlier, apoptosis and impaired clearance of apoptotic cells seem to be important in the development of CLE. KCs in CLE may be more susceptible to apoptosis. Toberer *et al.* [34] recently demonstrated enhanced expression in CLE lesions of Fas (CD95), a death receptor expressed on the cell surface that mediates the extrinsic pathway for apoptosis. TNF-related apoptosis-inducing ligand (TRAIL), a pro-apoptotic protein, is also enriched in the skin of CLE patients [35]. The KC receptor which mediates TRAIL apoptosis, TRAIL-R1, is also significantly enriched. Furthermore, TRAIL-R4, a TRAIL receptor which has anti-apoptotic properties, appears to be decreased. IFN-α enhances the expression of TRAIL by KCs, which underlines the importance of the interactions between KCs and IFN-α-producing pDCs in creating a pro-apoptotic environment [34, 35].

KCs found in CLE lesions express higher levels of receptors for IL-18, which is itself upregulated in lesions. KCs from CLE appear to be more susceptible to apoptosis when exposed to IL-18. When exposed to IL-18, these KCs also show higher levels of TNF-α expression. IL-12, a cytokine that has been shown to protect KCs from apoptosis and is upregulated in healthy skin in response to IL-18, is downregulated in KCs from CLE lesions [36].

In addition to their role as apoptotic cells, KCs are involved in a range of inflammatory stimuli crucial to the development of CLE. KCs, as stated previously, are responsible for the production of IL-1 and TNF-α, primary cytokines in the inflammatory cascade. These primary cytokines have a wide array of effects, including activation of antigen-presenting cells, induction of adhesion molecules, and recruitment of immune cells [9, 29].

KCs have also recently been found to express type III IFNs or IFN-λ. IFN-λ shares many functional similarities with type I IFNs, especially in terms of antiviral immunity. KCs produce high levels of IFN-λ1 in response to immunostimulatory nucleic acids, such as those from apoptotic cells. IFN-λ acts primarily on epithelial cells, and epithelial cells respond by producing pro-inflammatory cytokines like CXCL9, which enhances recruitment of immune cells (Fig. 1). In addition to being enriched at lesional sites, IFN-λ is also elevated in the serum of CLE patients with active lesions [37].

KC production of matrix metalloproteinases (MMPs) may also play a role in CLE. MMPs are enzymes which degrade components of the extracellular matrix and basement membrane. They are also implicated in cell growth, apoptosis, tissue repair, angiogenesis, inflammation, and immunity. While elevated levels of specific MMPs have been found in the serum of SLE, MMPs are less well characterized in CLE. Jarvinen *et al*. [38] found enhanced expression of MMP10 in basal KCs of CLE lesions. In animal models, elevated levels of MMP10 indicate impaired wound healing, and Jarvinen *et al.* [38] hypothesized that enhanced MMP10 may contribute to irregular matrix degradation in CLE. MMP7, which has been implicated in apoptotic pathways, is also enriched in basal KCs of CLE lesions. Despite the high levels of MMPs, inhibitors of MMPs, such as TIMP1, were not overexpressed, suggesting overall dysregulation of MMP activity. Interestingly, there was no difference between subtypes of CLE in terms of MMP expression [38].

KCs are responsible for production of high mobility group box 1 (HMGB1), a DNA binding protein found in most cells that also functions as a pro-inflammatory molecule extracellularly. HMGB1 is released by KCs in response to damage, such as UV radiation, or as part of apoptosis in CLE but not in healthy skin. It increases IFN-α production by pDCs (Fig. 1). Furthermore, HMGB1 may also be partially responsible for the impaired uptake of apoptotic KCs as it inhibits phosphatidylserine, which reduces phosphatidylserine-mediated phagocytosis. HMGB1 and its associated DNA may be taken up by immune cells and presented at lymph nodes to T and B cells, which may enhance autoimmunity [39].

KCs as well as endothelial cells (ECs) express adhesion molecules in response to damage and other stimuli. These adhesion molecules mediate migration of inflammatory immune cells. UV radiation is a known inducer of adhesion molecule expression and, in KCs, UV radiation specifically induces expression of ICAM-1. Elevated levels of soluble adhesion molecules have also been detected in CLE. Soluble ICAM-1 and VCAM-1 are elevated in the serum of both SCLE and SLE and are positively correlated with Ro/SSA and La/SSB antibodies. High levels of E-selectin, an adhesion molecule expressed only by ECs, were found only in DLE and not other subtypes of CLE [40].

### Endothelial cells

Cutaneous endothelial cells are responsible for production of a variety of factors which have been implicated in the pathogenesis of CLE. Interferon-induced guanylate-binding protein 1 (GBP1), a mediator of angiostasis in inflammation, is released by ECs in response to IFN-α and IFN-γ (Fig. 1). GBP1 appears to be a marker of pro-inflammatory microenvironments and functions to inhibit angiogensis and EC proliferation, spread, and migration. GBP1 has been detected in CLE lesions but not healthy skin. However, it is unknown whether GBP1 is merely a marker of inflammation or plays a role in CLE lesion formation and perpetuation [41].

Glycosaminoglycans (GAGs) have been implicated in CLE. GAGs produced by ECs and fibroblasts may function as pathogen-associated molecular patterns (PAMPs) and provide an inflammatory signal for sterile injury. GAGs are found in the dermal mucin that accumulates in CLE. Specifically, hyaluronic acid (HA) and chondroitin sulfate are found in the mucin deposits. GAGs can have both pro- and anti-inflammatory effects. For example, low molecular weight HA activates macrophages and dendritic cells and recruits certain immune cells and chondroitin sulfate promotes neutrophil activation. In contrast, high molecular weight HA appears to protect tissues from inflammation. UVB radiation, however, induces upregulation of genes expressing hyaluronidases and creates reactive oxygen species that may convert anti-inflammatory high molecular weight HA into pro-inflammatory fragments. HA fragments have been shown to activate immune cells such as dendritic cells and macrophages via Toll-like receptor (TLR)2 and TLR4. The accumulation of GAGs in dermal mucin may be acting to further perpetuate inflammation in CLE [42].

### Neutrophils and dendritic cells

Neutrophils have been implicated in the pathogenesis of CLE. Neutrophils defend against microbial invasion by forming neutrophil extracellular traps (NETs), structures composed of chromatin fibers and associated bactericidal proteins. Neutrophils undergo NETosis in response to microorganisms, cytokines, and other stimuli. NETs present double-stranded DNA and other factors such as LL37 that may play a part in inducing autoimmunity in LE. NET forming neutrophils are found in CLE lesions where they are concentrated at the dermoepidermal junction (DEJ), blood vessels, and adnexae. In addition, antibodies found in LE may increase NETosis in IFN-α primed neutrophils. Villanueva *et al*. [43] recently described a subset of reactive neutrophils found in patients with SLE. Named low-density granulocytes (LDGs), these neutrophils show upregulation of gene products involved in the inflammatory response, such as LL37 and others. LL37 is associated with NETs in the skin and microvesicles in serum of patients with CLE [43, 44]. It has been shown to convert endogenous DNA into potent agonists of TLRs on pDCs, which subsequently enhances type I IFN production (Fig. 1) [43]. LL37 also acts as a chemoattractant for a variety of immune cells and mediates the release of inflammatory cytokines from endothelial cells [43]. NET formation is enhanced in LDGs, which in turn increases externalization of putative autoantigens and immunostimulatory molecules [43]. Due to NET formation, presentation of putative autoantigens, and production of inflammatory signals, subsets of neutrophils unique to LE patients may be important in the pathogenesis of skin lesions.

pDCs, another component of the innate immune system, play an important role in the pathogenesis of CLE. pDCs are the main producers of type I IFNs, which mediate the inflammatory cascades in CLE. High numbers of type I IFN producing pDCs have been found in CLE lesions, while pDCs appear to be absent from normal skin. Elevated type I IFN production has been demonstrated by measuring expression of MxA, a type I IFN-inducible molecule. While pDCs normally produce type I IFNs in response to infection, it has been hypothesized that the high levels produced in CLE lesions may be in response to increased apoptosis, autoantibodies, and other immune cells, such as neutrophils, as mentioned above. Serum from LE patients combined with apoptotic cells from various lines has been shown to stimulate IFN-α production in pDCs *in vitro* [45]. pDCs are likely recruited to lesional sites due to inflammation and expression of chemokines, complement components, and adhesion molecules. For example, pDCs express high levels of L-selectin, and dermal endothelial cells in LE lesions express PNAd, a ligand for L-selectin. There are distinct subsets of pDCs in LE, ones that localize to the dermis and ones that localize to the DEJ. Dermal pDCs appear to mediate recruitment of Th1 T cells, while junctional pDCs recruit cytotoxic T cells [46, 47].

TLRs play a role in initiating pDCs as well as neutrophil activity. It has been shown in SLE that activation of pDCs through TLR7 and TLR9 by endogenous nucleic acids is a crucial part of the pathogenesis [48]. Activation through these TLRs leads to production of IFN-α and subsequent inflammatory events. Neutrophils also express these TLRs and are activated by TLR7 and TLR9 agonists. Lupus-prone (NZBxNZW)F1 mice develop chronic skin lesions resembling human CLE lesions after tape stripping. Antagonizing TLR7 and TLR9 results in a significantly more normal cutaneous response to tape stripping, suggesting that these TLRs may be required for initiation and maintenance of CLE lesions [48]. Antagonism of TLR7 and TLR9, however, does not impact the inflammatory cell infiltrate, suggesting that the stimuli for cell migration are separate from the stimuli for cytokine production. Guiducci *et al*. [48] hypothesize that while the initial reaction to cutaneous insult is normal in CLE, it is the prolongation of inflammatory stimuli and response that is responsible for the characteristic lesions in CLE. Immune complexes found at the DEJ and circulating autoantibodies may prolong stimulation of these receptors, leading to chronic cutaneous lesions. Impaired clearance of damaged and apoptotic KCs as described earlier may also be a source for prolonged activation of these TLRs.

### B cells

B cells act not only as antigen-presenting cells but also produce autoantibodies and secrete cytokines in autoimmune diseases such as CLE [49]. In DLE, specifically, it has been demonstrated that there is a higher density of B lymphocytes circulating peripherally and in lesional skin [50–52]. Interestingly, however, B cell-depleting therapies seem to lack efficacy in chronic cutaneous lupus erythematosus, such as DLE, while showing some effect in ACLE and SCLE [49]. Vital *et al.* [53] recently demonstrated a significant improvement after rituximab therapy in a proportion of patients with ACLE. In contrast, no patients with chronic cutaneous lupus erythematosus responded to B cell-depleting therapy, with some patients even experiencing flares in cutaneous disease. In light of these findings, the authors hypothesized that even if B cells are important in the initiation of cutaneous disease, they may not be essential for the perpetuation. As to why B cell-depleting therapy may provoke flaring of cutaneous disease, it may be that B cell-produced IL-10, an anti-inflammatory cytokine, is decreased after rituximab or that B-cell lysis may be pro-inflammatory [53]. Exactly how B cells participate in the pathophysiology of CLE has yet to be elucidated, but possible mechanisms include enhancement of autoimmune helper T cells and local secretion of autoantibodies, opsonization, and subsequent activation of the complement system [52, 54].

### Th1 cells

The inflammation in CLE appears to be predominantly Th1 mediated. Type I IFNs produced by pDCs recruit Th1 lymphocytes to CLE lesions by upregulating production of IP10/CXCL10 by KCs and other skin cells (Fig. 1). IP10/CXCL10 is a ligand for CXCR3, which is preferentially expressed by Th1 cells and therefore mediates the migration of Th1 cells to lesional sites. Ligands for CXCR3, like IP10/CXCL10, are the most abundantly expressed chemokine family members in CLE and correlate with the presence of pDCs and lymphocytes. pDC-produced IFN-α has been shown to induce Th1 differentiation and may also cause Th2 cells to convert to a Th1 phenotype. Th1 cells themselves produce IFN-γ, which perpetuates the upregulation of CXCR3 ligands and mediates downstream inflammatory effects responsible for lesion formation and persistence. IP10/CXCL10 and other Th1-specific ligands have also been shown to be potent antagonists of CCR3 expressed on Th2 cells. CXCR3-expressing lymphocytes are decreased in the blood of patients with CLE, further suggesting active recruitment of these cells to CLE lesions. Enhanced CCR5+ to CCR3+ lymphocyte ratio, indicating an enhanced Th1 to Th2 ratio, correlates with disease activity as measured by the Cutaneous Lupus Erythematosus Disease Area and Severity Index [27, 55, 56].

Th1-produced IFN-γ activates macrophages, enhances activity of cytotoxic T cells, mediates antiviral immune activity, and induces differentiation of naive T cells into Th1 cells. IFN-γ is also able to upregulate its own expression [57]. The role of IFN-γ in development and persistence of CLE lesions is also supported by findings in transgenic mice overexpressing IFN-γ in the epidermis. These mice develop a lupus-like syndrome with autoantibodies and CLE-like skin lesions. IFN-γ-induced skin lesions from this model and CLE skin lesions appear to share multiple characteristics, such as similar dermal infiltrate, KC expression profile, and enhanced apoptosis. Furthermore, exogenously administered IFN-γ in humans has been reported to cause LE with prominent CLE lesions. Additionally, depletion of CD4+ T cells with monoclonal antibodies has been shown to result in significantly decreased disease activity in severe CLE, highlighting the crucial role of helper T cells in the pathogenesis of this disease [58].

### Th17 cells

The role of Th17 cells is not as clear as those of other immune cells. Oh *et al*. [59] showed that expression of IL-17A, an isoform of IL-17, positively correlated with expression of IFN-α in CLE lesions compared with psoriatic skin lesions. IL-17A was presumably expressed by Th17, though other cell types are known to produce this cytokine. IL-6, a cytokine involved in inflammation and differentiation of Th17 cells, is likely responsible in part for Th17 activity in CLE. The investigators showed elevated levels of IL-6 in lesional skin. While the source of the IL-6 was not determined, fibroblasts, macrophages, endothelial cells, T cells, and B cells are known to produce this cytokine in response to IL-1, TNF-α, and IFNs [59]. Tanasescu *et al*. [60] also found elevated expression of IL-17 in the skin of patients with CLE. IL-17A was elevated in the serum of patients with DLE and SLE, but not in SCLE. IL-17 F, however, was elevated in SCLE serum. Elevated IL-17A in the serum correlated with IL-17+ lymphocytes in the cutaneous inflammatory infiltrate only in DLE. Interestingly, the presence of anti-Ro/SSA antibodies in the SCLE serum correlated with enhanced presence of IL-17A+ lymphocytes in the skin. There may be an association between exposure to the Ro antigen and expression of IL-17A through activation of Th17 cells by dendritic cells [60].

In contrast to these findings, however, Jabbari *et al*. [61] found very little evidence of Th17 cells in DLE. The investigators demonstrated enrichment of Th1 cells and absence of Th17 cells in DLE lesions. IFN-γ regulated genes were upregulated whereas IL-17 genes did not show increased expression. Whether or not Th17 cells are significant players in CLE has yet to be determined.

### Regulatory T cells

Tregs function to inhibit inflammation by secreting inhibitory cytokines such as IL-10 and transforming growth factor-β and by suppressing other T cells through direct contact. Treg numbers are reduced in CLE lesions. However, while in SLE a decrease in circulating Tregs has been observed, in CLE this does not appear to be the case. Interestingly, the Treg reduction in lesional skin of CLE patients is not observed in other inflammatory skin diseases such as atopic dermatitis, psoriasis, and lichen planus. To explore the etiology of the reduction of Tregs, Franz *et al*. [62] tested the response of Tregs from CLE patients to Fas ligand (CD95L), a molecule known to induce apoptosis. It has been previously hypothesized that low numbers of Tregs may be due to increased susceptibility to apoptosis in response to this ligand. Franz *et al*., however, found no evidence of increased susceptibility to apoptosis in patient-derived Tregs. Furthermore, the investigators found no difference between CLE-derived and control-derived Tregs in their ability to suppress conventional T cell proliferation.

### Cytotoxic CD8+ T cells

Cytotoxic CD8+ T cells (CTLs) contribute to the inflammatory infiltrate found in CLE lesions. These cells express granzyme B, a serine protease which is able to prime cells for apoptosis by activating caspases (Fig. 1). Granzyme B expression is associated with the DEJ infiltrate in CLE and positively correlates with expression of IFN-α. IFN-α, presumably produced by pDCs, is able to stimulate development of CTLs, enhance their cytotoxicity, and upregulate MHC class I molecules in tissues, thus making those tissues targets for this type of T cell. Additionally, increased activity of CTLs appears to be more associated with the destructive and scarring types of CLE, such as DLE. Given their presence in CLE lesions and production of pro-apoptotic molecules, CTLs appear to play a role in CLE, though the level of their significance is not certain [63, 64].

### Invariant natural killer T cells

Human invariant natural killer T cells (iNKTs) are a subtype of T lymphocytes which release Th1- and Th2-like cytokines, including IFN-γ, TNF-α, IL-4, and IL-10. Due to the array of cytokines they are able to produce, iNKTs have a broad spectrum of functionality and are able to promote or suppress inflammation. iNKTs respond to lipid antigens presented by CD1d, in contrast to other T cells which respond to peptides presented by MHC. CD1d is an antigen-presenting molecule expressed on epithelial cells like KCs, dendritic cells, and B cells. It has been shown that trauma upregulates expression of CD1d on KCs. Lower numbers of circulating iNKTs are correlated with more severe disease activity of CLE, and the amount of immunosuppressive therapy does not seem to influence circulating iNKTs. Hofmann *et al*. [65] demonstrated enrichment of iNKTs in the lesions of all types of CLE and hypothesized that circulating deficiency of iNKTs may be due to active recruitment and migration to lesional sites. The investigators also found expression of IFN-γ by iNKTs in lesions. Thus, iNKTs may contribute to the inflammatory microenvironment of CLE lesions.

## Conclusion

CLE is a heterogeneous disease with a pathophysiology that has yet to be fully characterized. The current understanding of CLE risk factors, markers, and cellular components can aid in prognostics and the development of novel therapies. To this end, additional research is needed to better clarify the mechanism of this disease.

## Abbreviations

ACLE: Acute cutaneous lupus erythematosus
CLE: Cutaneous lupus erythematosus
CTL: Cytotoxic CD8+ T cell
DEJ: Dermoepidermal junction
DLE: Discoid lupus erythematosus
EC: Endothelial cell
GAG: Glycosaminoglycan
HA: hyaluronic acid
IFN: interferon
Ig: Immunoglobulin
IL: Interleukin
iNKT: Invariant natural killer T cell
KC: Keratinocyte
LDG: Low-density granulocyte
LE: Lupus erythematosus
MHC: Major histocompatibility complex
MMP: Matrix metalloproteinase
NET: Neutrophil extracellular trap
pDC: Plasmacytoid dendritic cell
SCLE: Subacute cutaneous lupus erythematosus
SLE: Systemic lupus erythematosus
SNP: Single-nucleotide polymorphism
TLR: Toll-like receptor
TNF: Tumor necrosis factor
Treg: Regulatory T cell
UV: Ultraviolet
